# Few-Layered Black Phosphorene as Hole Transport Layer for Novel All-Inorganic Perovskite Solar Cells

**DOI:** 10.3390/ma18020415

**Published:** 2025-01-17

**Authors:** Shihui Xu, Lin Yang, Zhe Wang, Fuyun Li, Xiaoping Zhang, Juan Zhou, Dongdong Lv, Yunfeng Ding, Wei Sun

**Affiliations:** 1 Hainan Engineering Research Center of Tropical Ocean Advanced Optoelectronic Functional Materials, Hainan International Joint Research Center of Marine Advanced Photoelectric Functional Materials, Key Laboratory of Laser Technology and Optoelectronic Functional Materials of Hainan Province, Key Laboratory of Functional Materials and Photoelectrochemistry of Haikou, College of Chemistry and Chemical Engineering, Hainan Normal University, Haikou 571158, China; xushihui10@163.com (S.X.); yanglin19981223@163.com (L.Y.); 15596056261@163.com (Z.W.); sindy2162003@163.com (J.Z.); 2 Engineering Research Center of Environmentally-Friendly Functional Materials, Ministry of Education, Huaqiao University, Xiamen 361021, China; 3 College of Physics and Electronic Engineering, Hainan Normal University, Haikou 571158, China; 202313085408015@hainnu.edu.cn (F.L.); hsgc16637901209@163.com (D.L.); 18943162113@163.com (Y.D.)

**Keywords:** black phosphorus, hole transport layer, CsPbBr_3_, perovskite, solar cells

## Abstract

The CsPbBr_3_ perovskite exhibits strong environmental stability under light, humidity, temperature, and oxygen conditions. However, in all-inorganic perovskite solar cells (PSCs), interface defects between the carbon electrode and CsPbBr_3_ limit the carrier separation and transfer rates. We used black phosphorus (BP) nanosheets as the hole transport layer (HTL) to construct an all-inorganic carbon-based CsPbBr_3_ perovskite (FTO/c-TiO_2_/m-TiO_2_/CsPbBr_3_/BP/C) solar cell. BP can enhance hole extraction capabilities and reduce carrier recombination by adjusting the interface contact between the perovskite and the carbon layer. Due to the coordination of the energy structure related to interface charge extraction and transfer, BP, as a new type of hole transport layer for all-inorganic CsPbBr_3_ solar cells, achieves a power conversion efficiency (PCE) that is 1.43% higher than that of all-inorganic carbon-based CsPbBr_3_ perovskite solar cells without a hole transport layer, reaching 2.7% (Voc = 1.29 V, Jsc = 4.60 mA/cm^2^, FF = 48.58%). In contrast, the PCE of the all-inorganic carbon-based CsPbBr_3_ perovskite solar cells without a hole transport layer was only 1.27% (Voc = 1.22 V, Jsc = 2.65 mA/cm^2^, FF = 39.51%). The unencapsulated BP-based PSCs device maintained 69% of its initial efficiency after being placed in the air for 500 h. In contrast, the efficiency of the PSC without HTL significantly decreased to only 52% of its initial efficiency. This indicates that BP can effectively enhance the PCE and stability of PSCs, demonstrating its great potential as a hole transport material in all-inorganic perovskite solar cells. BP as the HTL for CsPbBr_3_ PSCs can passivate the perovskite interface, enhance the hole extraction capability, and improve the optoelectronic performance of the device. The subsequent doping and compounding of the BP hole transport layer can further enhance its photovoltaic conversion efficiency in PSCs.

## 1. Introduction

Organic–inorganic hybrid perovskites exhibit an excellent photovoltaic performance and have become the most popular photovoltaic materials in recent years. Since their first preparation in 2009, the PCE of organic–inorganic hybrid PSCs has significantly increased from 3.8% to 26.7% [1,2,3,4,5]. Although organic–inorganic hybrid PSCs have developed rapidly, there are still some issues in practical applications, such as methylammonium lead iodide (MAPbI_3_), and they degrade quickly when exposed to light, humidity, oxygen, or high-temperature environments, exhibiting significant instability [6,7] and limiting the practical application of organic–inorganic hybrid perovskite solar cells. The method of using Cs^+^ to replace organic cations to enhance the stability and thermal stability of PSCs has been proven to be effective [8,9].

All-inorganic lead cesium halide (CsPbX_3_) perovskites have garnered widespread attention as light-absorbing materials due to their excellent thermal stability (thermal stability > 400 °C) and good optoelectronic properties [10,11]. Currently, there are mainly four types of CsPbX_3_ solar cells: CsPbI_3_ [12], CsPbBr_3_, CsPbI_2_Br [13,14], and CsPbIBr_2_ [15]. However, CsPbI_3_ perovskite struggles to maintain its ideal black cubic α phase at room temperature and easily transforms into the yellow orthorhombic δ non-perovskite phase, leading to a decline in its optoelectronic performance [16]. Br^−^ possesses enhanced effective tolerance factors and lower-phase transition temperatures [17], which contribute to the greater stability of the black cubic α-phase at room temperature. Therefore, compared to CsPbI_2_Br and CsPbIBr_2_, CsPbBr_3_ perovskite exhibits better environmental stability. Additionally, CsPbBr_3_ is easy to prepare in ambient conditions without the need for humidity control, has a high carrier mobility, and is one of the most promising inorganic light absorbers [18]. However, the large optical bandgap of CsPbBr_3_ (~2.3 eV) results in a poor light absorption capability within the perovskite film of solar cells. Additionally, the significant energy barrier and high defect states at the CsPbBr_3_/Carbon interface can lead to poor charge extraction and severe carrier recombination, further diminishing the performance of the final device. Li et al. [19] used zinc phthalocyanine to composite CsPbBr_3_ quantum dots (ZnPc/CsPbBr_3_ QDs) for the synergistic modification of the CsPbBr_3_/Carbon interface in CsPbBr_3_ PSCs. This approach passivated the interfacial trap states, improved the contact at the CsPbBr_3_/Carbon interface, and optimized the energy level arrangement at the interface. As a result, it enhanced carrier extraction and suppressed carrier recombination, leading to a maximum efficiency of 10.20% for the PSCs, with long-term stability exceeding 6 months. Therefore, adjusting the interfacial contact of the perovskite, passivating defects, and modulating the energy level difference between CsPbBr_3_ and the Carbon layer are key to improving CsPbBr_3_ PSCs.

Black phosphorus (BP), as a two-dimensional layered material, possesses characteristics such as high theoretical mobility, a tunable direct bandgap, bipolar properties, and simple fabrication. At room temperature, its carrier mobility can reach approximately 1000 cm^2^/Vs [20]. The direct bandgap property of BP is related to the number of layers, ranging from 0.3 eV (bulk) to 2 eV (monolayer) [21]. BP can serve as a dual-function interface modifier, reducing perovskite crystallization and the defect density in the electron transport layer (ETL)/perovskite, thereby enhancing the charge carrier transport [22,23]; it also strengthens hole extraction in perovskite/HTL [24]. Liu et al. [25] used BP as the ETL in organic photovoltaics (OPVs). When the optimal thickness of BP is 10 nm, a cascading band structure can be formed in the OPV, facilitating an electron transfer and enhancing the device’s power conversion efficiency. Gu et al. [26] utilized black phosphorus quantum dots (BPQDs) mixed with SnO_2_ as a mixed ETL for (FAPbI_3_)_0.97_(MAPbBr_3_)_0.03_ perovskite solar cells. The strong interaction between BPQDs and SnO_2_ not only alters the inherent defects of the SnO_2_ layer, improving the carrier transport capability, but also suppresses the oxidation of BPQDs. BP has been widely studied as an electron transport layer in PSCs, but research on its role as a hole transport layer in CsPbBr_3_ PSCs is relatively limited. Muduli et al. [27] were the first to use two-dimensional BP nanosheets as the HTL in MAPbI_3_ perovskite solar cells, demonstrating that BP nanosheets have a strong hole extraction capability. When BP nanosheets were used solely as the HTL, the device achieved a PCE of 7.88%, an improvement over the 4% of devices without a hole transport material (HTM). Dong et al. [28] used few-layered 2D BP nanosheet-doped poly(triarylamine) (BP:PTAA) as a HTL, significantly improving the charge extraction at the perovskite–HTL interface and reducing the energy barrier. Liu et al. [29] introduced Spiro-OMeTAD:BPQDs as the HTL for Cs_0.05_(FA_0.83_MA_0.17_)_0.95_Pb(I_0.83_Br_0.17_)_3_ perovskite solar cells, and the incorporation of BPQDs significantly enhanced the hole mobility of Spiro-OMeTAD, facilitating hole transport and improving the device performance. Therefore, BP is a viable option as a HTL for perovskite solar cells and using BP as the HTL for CsPbBr_3_ PSCs can passivate the perovskite interface, enhance the hole extraction capability, and improve the optoelectronic performance of the device. Moreover, BPQDs are proposed as effective seed-like sites to modulate the nucleation and growth of CsPbIBr perovskite crystalline thin layers, allowing an enhanced crystallization and remarkable morphological improvement. Huang et al. [30] utilized the addition of BP chloroform to the Pb precursor solution of perovskite, which was then mixed with the bromide precursor solution to obtain a CsPbBr_3_-BP heterostructure film with strong bonding, exhibiting good optical and electronic tunability. Gong et al. [22] injected BP as an additive into the precursor solution of perovskite to prepare a BPQDS/CsPbI_2_Br core–shell structure, which enhanced the stability of the CsPbI_2_Br crystals, suppressed the oxidation of BPQDS, and effectively improved the stability of the solar cells, providing insights for achieving efficient and stable inorganic perovskite solar cells. However, in carbon-based CsPbBr_3_ perovskite solar cells, the potential of BP as a HTL between CsPbBr_3_ and carbon has yet to be explored. By using BP as the HTL between CsPbBr_3_ and carbon, and through the interaction between BP and CsPbBr_3_, investigating the crystallization and phase stability of CsPbBr_3_ could further enhance the optoelectronic performance of the devices.

**Figure 1:**
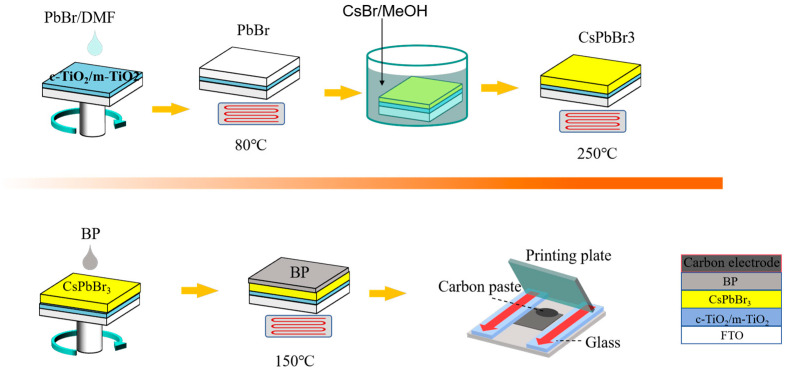
Preparation process of the FTO/c-TiO_2_/m-TiO_2_/CsPbBr_3_/BP/C device.

In this paper, a two-step method is employed to synthesize CsPbBr_3_ thin films, using few-layer BP nanosheets as the HTL layer for CsPbBr_3_ perovskite-based solar cells. Fully inorganic PSCs with the structure FTO/c-TiO_2_/m-TiO_2_/CsPbBr_3_/BP/C are fabricated, and the preparation process is illustrated in Figure 1. By optimizing the battery preparation conditions, a maximum PCE of 2.70% was achieved (Voc = 1.29 V, Jsc = 4.60 mA/cm^2^ and FF = 48.58%), higher than the CsPbBr_3_ devices without HTL which had a PCE of 1.27% (Voc = 1.22 V, Jsc = 2.65 mA/cm^2^, FF = 39.51%). After being exposed to air for 500 h, the unencapsulated BP-based PSC device still retained 69% of its initial PCE. In contrast, the PSC without HTL showed significant degradation, with a 48% loss of the initial PCE.

## 2. Materials and Methods

### 2.1. Materials

Fluorine-doped tin oxide conductive glass (FTO, ≤10 Ω, 2.5 × 2.5 cm^2^) was purchased from Advanced Election Technology Co., Ltd. (Yingkou, China). Tetra-isopropyl titanate (99.99%), TiO_2_ paste (18NR-T Titania Paste), and lead bromide (99.99%) were obtained from Youxuan Technology. Anhydrous ethanol, N, N-dimethylformamide (DMF), methanol, isopropanol (IPA), and cesium bromide (99.99%) were sourced from Sinopharm Chemical Reagent Co., Ltd. (Shanghai, China). Concentrated hydrochloric acid, black phosphorus nanosheet dispersion (0.2 mg/mL, solvent ethanol), and conductive carbon paste were purchased from Jiangsu Xianfeng Nanomaterials Technology Co., Ltd. (Nanjing, China).

### 2.2. Device Fabrication

The FTO was ultrasonically cleaned in cleaning powder, acetone, and isopropanol for 20 min each, and then stored in anhydrous ethanol for later use. When needed, the conductive glass was dried with a hairdryer. First, 34 μL of 2 M HCl solution was added to 2 mL of anhydrous ethanol, then 254 μL of titanium isopropoxide was added. This was then stirred at room temperature for 12 h to obtain a dense TiO_2_ (c-TiO_2_) solution. The mesoporous TiO_2_ (m-TiO_2_) solution was prepared by evenly mixing commercially available TiO_2_ slurry with anhydrous ethanol in a mass ratio of 5:1. Next, 40 μL of c-TiO_2_ solution was applied onto the FTO using a spin coater at a speed of 3000 rpm for 30 s to form a c-TiO_2_ layer, and then the coated glass was annealed at 500 °C for 30 min. The m-TiO_2_ layer was also prepared using the spin-coating method. A volume of 40 μL of m-TiO_2_ solution was spun at a speed of 3000 rpm for 30 s. After drying under an infrared lamp, the material was sintered at 500 °C for 30 min.

The preparation of the perovskite film is as follows: First, 1 M PbBr_2_ DMF solution was spin-coated onto m-TiO_2_ at a speed of 2000 rpm for 30 s, followed by annealing at 80 °C for 30 min. Then, the PbBr_2_ film was immersed in a 0.7 M CsBr methanol solution for 30 min to form a CsPbBr_3_ film. After washing the CsPbBr_3_ film in isopropanol, it was annealed on a heating platform at 250 °C for 5 min. Preparation of the BP hole transport layer: The prepared CsPbBr_3_ film was placed on a spin coater, and 30 μL of BP dispersion was spin coated at 1500 rpm for 30 s. Afterward, it was annealed at 150 °C for 30 min. Finally, conductive carbon paste was applied onto the BP film using a scraper and annealed on a heating plate at 120 °C for 15 min to obtain the carbon electrode.

### 2.3. Characterization

The morphology and structure of PSCs, the electron transport layer, and BP were characterized using a scanning electron microscope (SEM, JSM-7100, Shimadzu, Kyoto, Japan) at 200 kV. The crystal structure of the perovskite layer and the perovskite/BP layer films was analyzed using an X-ray diffractometer (XRD, D8 Advance, Bruker, Bremen, Germany) with Cu-Kα radiation (λ = 1.5404 Å) and this test had a scan speed of 2°/min and a scan range of 10–80°. The steady-state transient fluorescence spectra (PL) were tested using a fluorescence spectrophotometer (Hitachi F-7000, Tokyo, Japan) with a 520 nm laser excitation source. [29] The time-resolved photoluminescence spectroscopy (TRPL) test was conducted using a 520 nm excitation light source on the Horiba FluoroMax + high-sensitivity integrated fluorescence spectrometer (Kyoto, Japan). Under a light intensity of 1.5 G (100 mW cm^−2^) provided by the 3A level solar simulator (Newport 94023A, 450 W, Irvine, CA, USA), the photo current density–voltage (J-V) curves were recorded using a Keithley 2400 digital source meter (Cleveland, OH, USA). The light intensity was calibrated using a silicon reference cell equipped with a power meter (NREL). Electrochemical impedance spectroscopy (EIS) was conducted using an electrochemical workstation (CHI660E, Chenhua, Shanghai, China) and measurement was a Voc test on the PSC by an electrochemical workstation, obtaining bias, and then EIS testing at the corresponding bias in dark.

## 3. Results

### 3.1. Characterization of SEM and XRD

**Figure 2:**
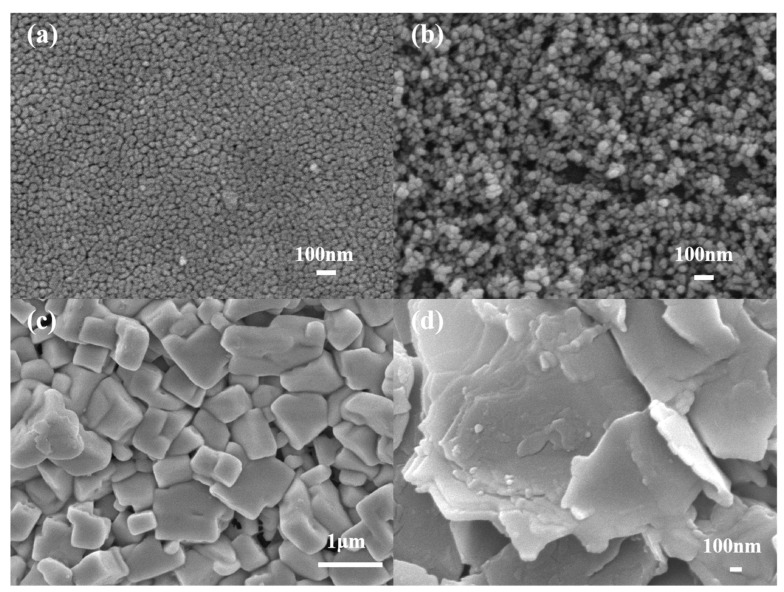
SEM images: (**a**) c-TiO_2_ layer, (**b**) m-TiO_2_ layer, (**c**) CsPbBr_3_ layer, and (**d**) BP on the surface of CsPbBr_3_.

**Figure 3:**
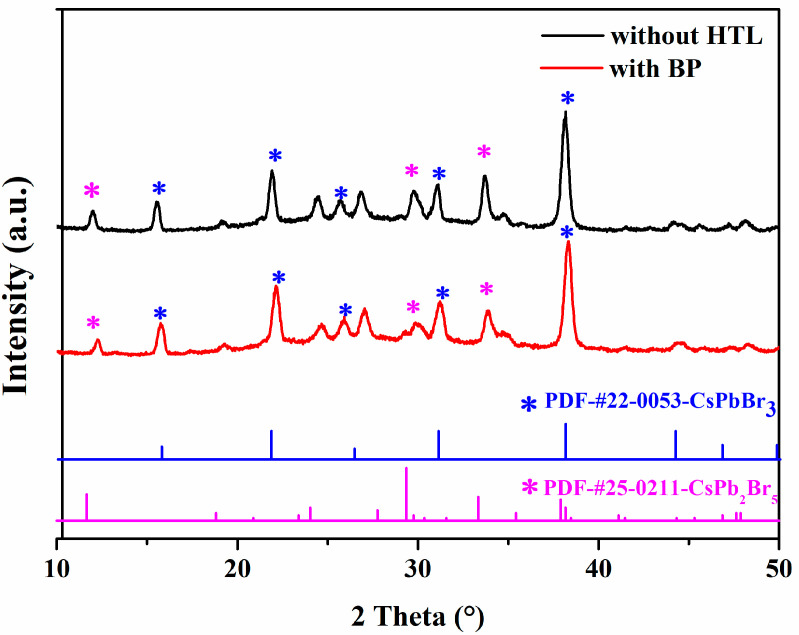
XRD patterns of CsPbBr_3_ films without BP HTL and CsPbBr_3_ films with BP.

Figure 2 shows the SEM images of the c-TiO_2_ layer, m-TiO_2_ layer, perovskite layer, and BP prepared under optimal conditions. From Figure 2a, we can see that the c-TiO_2_ layer consists of densely packed nanoparticles, forming a smooth and compact structure. This smooth, dense layer can effectively block holes, suppress charge recombination, reduce the leakage current, and prevent battery short circuits. From Figure 2b, it is seen that the m-TiO_2_ layer has many nanopores, which will serve as a good carrier for the injection of perovskite. From Figure 2c, it is seen that the CsPbBr_3_ film is smooth, dense, and has good crystallinity. Figure 2d shows the SEM image of BP on the perovskite surface. It is clearly observable that a layer of BP, which is a nanometer-scale two-dimensional layered structure, is coated on top of the perovskite. To investigate the effect of BP as a hole transport layer on the crystal structure of perovskite, we conducted XRD tests on CsPbBr_3_ films and BP. The results, shown in Figure 3, reveal four characteristic peaks centered at 15.8°, 21.9°, 25.6°, 31.1°, and 38.1°, which correspond to the main lattice planes (100), (110), (111), (200), and (210) of the CsPbBr_3_ phase, respectively. Additionally, three characteristic peaks of 12.0°, 29.7°, and 33.7° correspond to (002), (213), and (210) of the CsPb_2_Br_5_ phase. This is a common phenomenon in the two-step synthesis of CsPbBr_3_ films [31]. The characteristic peaks of the perovskite film are clear and sharp, indicating the successful formation of the CsPbBr_3_ crystal structure. The diffraction peak intensity of the perovskite film with BP about the CsPbBr_3_ phase is stronger, with sharper peaks at 25.6° and 31.1°. Among them, the three characteristic peaks of the CsPb_2_Br_5_ phase were decreased by 12.0°, 29.7°, and 33.7°. However, there is no peak shift, indicating that BP, as the HTL, did not enter the perovskite crystal, but instead reduced the interface defects of CsPbBr_3_ and improved its crystallinity.

### 3.2. Preparation and Performance Regulation of CsPbBr_3_ Solar Cells Based on BP HTL

**Figure 4:**
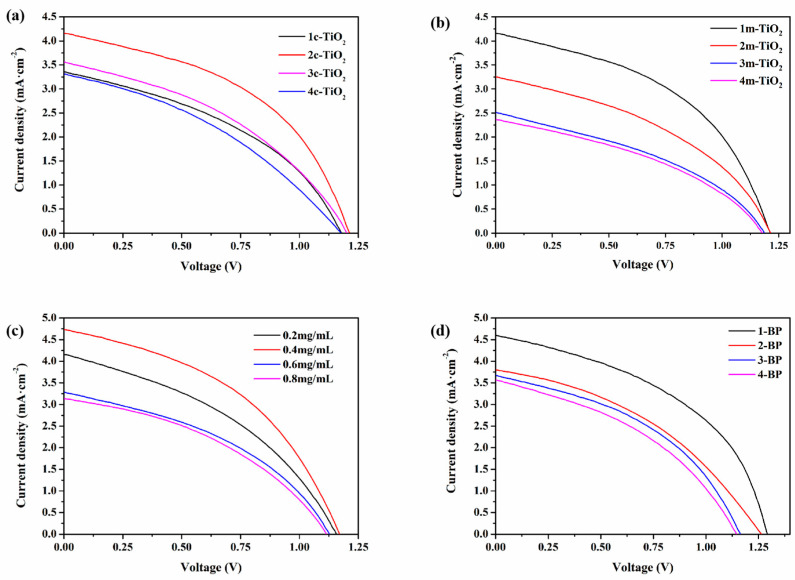
(**a**) J-V curves of CsPbBr_3_ PSC with different numbers of c-TiO_2_ spin-coating; (**b**) J-V curves of CsPbBr_3_ PSC with different numbers of m-TiO_2_ spin-coating; (**c**) J-V curves of CsPbBr_3_ PSC with different BP concentrations; (**d**) J-V curves of CsPbBr_3_ PSC with different numbers of BP spin-coating.

To investigate the optimal optoelectronic performance of CsPbBr_3_ PSCs, the preparation conditions of the batteries were optimized. Figure 3 shows the J-V curves of CsPbBr_3_ PSCs under different preparation conditions, while Table 1 lists the photovoltaic parameters of CsPbBr_3_ PSCs under the corresponding conditions: open-circuit voltage (Voc), short-circuit current (Jsc), fill factor (FF), and power conversion efficiency (PCE). First, we will explore the impact of the number of spin-coating cycles of c-TiO_2_ on the optoelectronic performance of the device. The spin-coating cycles of c-TiO_2_ are set to one, two, three, and four times, and J-V characteristic curve tests were conducted. As shown in Figure 4a and Table 1, when the spin-coating cycles of c-TiO_2_ are set to two, the battery achieves the best PCE of 2.31%. Next, using the optimized values for the spin-coating count of c-TiO_2_, the effect of m-TiO_2_ spin-coating the optoelectronic performance of the device was investigated, with the spin-coating counts for m-TiO_2_ being set to one, two, three, and four. From Figure 4b and Table 1, it can be observed that when the spin-coating count of m-TiO_2_ is one, the battery achieves the best optoelectronic efficiency. This may be because the permeability of the m-TiO_2_ layer decreases as the spin-coating count increases. Therefore, when the spin-coating count of m-TiO_2_ is one, it can improve the interface contact between the electron transport layer (ETL) and the perovskite layer, while having a minimal impact on the light absorption performance of the CsPbBr_3_ perovskite layer. Next, using the optimized values for ETL components, the effect of the BP concentrations was investigated, with the BP concentrations being set at 0.2, 0.4, 0.6, and 0.8 mg/mL. From Figure 4c and Table 1, it can be observed that when the BP concentration is 0.4 mg/mL, the device achieves the optimal photoelectric efficiency of 2.42%. Finally, using the optimized values for the ETL components and BP concentration, the effect of the BP spin-coating times on the device’s optoelectronic performance was studied, with the spin-coating times for BP being set at 0.2, 0.4, 0.6, and 0.8 mg/mL. According to Figure 4d and Table 1, it can be observed that when the spin-coating time for BP is one, the device achieves the best optoelectronic efficiency of 2.70%.

### 3.3. Photovoltaic Characteristics of CsPbBr_3_ Solar Cells Based on BP HTL

**Figure 5:**
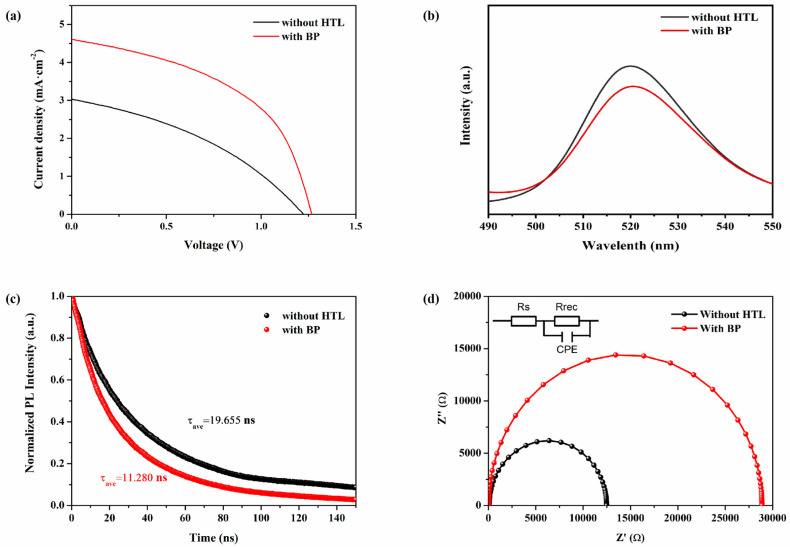
(**a**) J-V curves of CsPbBr_3_ PSCs with and without HTL under a light intensity of 100 mW/cm^2^; (**b**) PL of CsPbBr_3_ films without HTL and CsPbBr_3_/BP films; (**c**) TRPL of CsPbBr_3_ films without HTL and CsPbBr_3_/BP films; (**d**) Impedance plots of CsPbBr_3_ PSCs with and without HTL, using 0.4 mg/mL BP as HTL.

**Figure 6:**
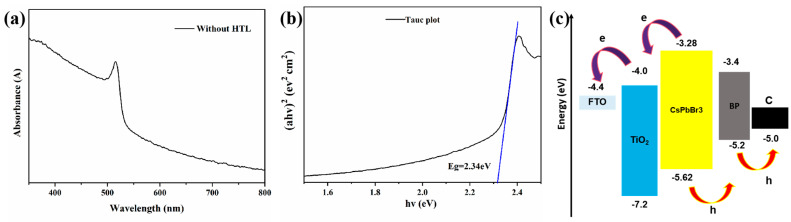
(**a**) UV–vis spectra of the CsPbBr_3_ film without HTL, (**b**) Tauc plot, (**c**) Band alignment of perovskite solar cells with BP.

Figure 5a shows the J-V characteristics of CsPbBr_3_ PSCs using 0.4 mg/mL BP as the HTL and those without HTL, under illumination at an intensity of 100 mW/cm^2^. The photovoltaic parameters are listed in Table 2. The PCE of CsPbBr_3_ PSCs without HTL is 1.27%, with Voc at 1.22 V, Jsc at 2.65 mA/cm^2^, and FF at 39.51%. In contrast, PSCs using 0.4 mg/mL BP as the HTL exhibit a higher Voc of 1.29 V, Jsc of 4.60 mA/cm^2^, and FF of 45.52%, thereby increasing the PCE to 2.70%. To further investigate the principle of BP as a HTL in enhancing the optoelectronic performance of CsPbBr_3_ PSCs, PL spectroscopy and TRPL testing were conducted on perovskites with and without a HTL. Steady-state light can reflect the recombination and separation of electrons and holes in perovskite materials. When a HTL is present on the perovskite layer, the layer is excited by light, causing the separation of electrons and holes, with holes transferring to the HTL, leading to PL quenching. The more pronounced the PL quenching effect, the lower the intensity of the PL emission peak, indicating that the charge transfer at the interface occurs more rapidly [32]. From Figure 5b, it is seen that CsPbBr_3_ without a hole transport layer exhibits a strong fluorescence peak, indicating that the recombination of electrons and holes is quite severe in the absence of a hole transport layer. The PL intensity of the perovskite film with BP as the HTL is about 1.15 times lower than that of the perovskite film without a HTL. This suggests that the degree of recombination in the perovskite with BP as the HTL is smaller, and it also proves that BP has good hole extraction capabilities, effectively extracting holes from the light absorption layer and significantly reducing the recombination of electrons and holes. Figure 5c compares the TRPL spectra of perovskite films with and without BP as the HTL. Table 3 lists the TRPL spectral parameters for CsPbBr_3_ films without the HTL and CsPbBr_3_ films with BP as the HTL. The TRPL data were fitted using biexponential decay with a fast decay lifetime (τ_1_) and slow decay lifetime (τ_2_): I(t) = A_1_ exp(−t/τ_1_) + A_2_ exp(−t/τ_2_). Among them, τ_1_ mainly represents the non-radiative recombination caused by grain boundaries and surface trap states, while τ_2_ is attributed to the radiative recombination of free-charge carriers. The τ_1_ and τ_2_ for FTO/CsPbBr_3_ are 4.931 ns and 23.961 ns, respectively. In comparison, for FTO/CsPbBr_3_/BP, τ_1_ and τ_2_ decrease to 4.055 ns and 14.337 ns, respectively. This indicates that BP has a high charge extraction capability, which can enhance the hole extraction ability of the device, allowing carriers to transfer more effectively from the perovskite layer to the HTL. This reduces the defect density and suppresses the charge recombination at the perovskite/HTL interface, thereby improving the photovoltaic performance of PSCs. Consequently, the use of BP as the HTL significantly enhances the Jsc and FF of CsPbBr_3_ PSCs, consistent with the measurement results shown in Figure 5a. Figure 5d shows the electrochemical impedance spectra (EIS) of CsPbBr_3_ PSC devices with and without HTL, using 0.4 mg/mL BP as the HTL. Here, Rs represents the transfer resistance within the entire PSC, Rrec corresponds to the internal charge recombination resistance indicated by the semicircle, and CPE refers to the phase angle element. Table 4 lists the EIS parameters of CsPbBr_3_ PSCs with 0.4 mg/mL BP as the HTL and without HTL. In the devices of FTO/c-TiO_2_/m-TiO_2_/CsPbBr_3_/C and FTO/c-TiO_2_/m-TiO_2_/CsPbBr_3_/BP/C, Rrec is the dominant factor. The Rrec value of BP as the HTL in CsPbBr_3_ PSC devices is 28,862 Ω, which is significantly higher than that of CsPbBr_3_ PSC devices without an HTL (Rrec = 12,397 Ω). Meanwhile, the Rs of the without-HTL PSC was 91.37 Ω, which was significantly smaller than the Rs of with-BP PSCs. This indicates that BP notably inhibits the non-radiative recombination, improves the charge transmission efficiency, and passivates the device defects. This may be due to the interaction between P in BP and Pb in perovskite, which leads to a reduction in defects and an increase in the Rrec value. This effectively suppresses charge recombination, facilitating the extraction of charge carriers in the device, thereby enhancing the FF and Jsc. Figure 6a,b is the UV spectra and Tauc plot of the perovskite thin film without a hole transport layer. The data indicate that the bandgap of CsPbBr_3_ is 2.34 eV, which is consistent with the values reported in the literature for CsPbBr_3_ [32]. With the above-calculated energy bands and the data from the reference [23], the energy band diagram of the CsPbBr_3_ PSC can be drawn, which is shown in Figure 6c. Here, we can see that the maximum valence band (EVB) of the CsPbBr_3_ layer film is −5.62 V, while the maximum valence band of BP is −5.2 V. As a hole transport layer (HTL), BP reduces the energy offset for hole extraction, which facilitates a faster hole transfer and suppresses hole accumulation at the interface, thereby improving the device’s photoelectric conversion efficiency.

### 3.4. Stability Testing

**Figure 7:**
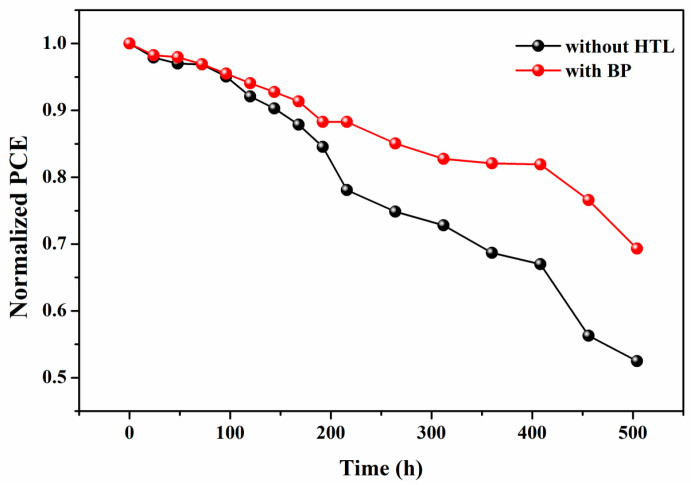
Under ambient air conditions at room temperature and relative humidity ranging from 25% to 65%. Comparison of the stability of unencapsulated CsPbBr3 PSC with BP as HTL and without HTL.

The stability of CsPbBr_3_ PSCs is a key factor for their commercialization. Figure 7 compares the PCE stability tests of PSCs with and without BP HTL. After being stored in air at a relative humidity (RH) of 25–65% and a temperature of 25 °C for 500 h, the unencapsulated optimal PSCs retained 69% of their initial PCE. In contrast, the PSCs without the HTL showed significant degradation, losing 48% of their initial PCE. This indicates that BP is in close contact with the perovskite layer and the carbon layer. Due to the protection provided by the carbon layer, the degradation rate of BP is slower, which enhances the air stability of the PSCs. Additionally, incorporating BP into the perovskite layer can passivate the Pb^0^ defects in the perovskite layer, thereby improving the stability of the device, which is consistent with previous theoretical studies [21].

## 4. Conclusions

In summary, BP can effectively adjust the interface contact of CsPbBr_3_/Carbon, reduce charge recombination, and facilitate the extraction of interface charge carriers. At the same time, BP, as a hole transport layer, enhances the hole extraction capability of CsPbBr_3_ PSCs, leading to improved Jsc and FF. Ultimately, this significantly enhances the optoelectronic performance of all-inorganic CsPbBr_3_ PSCs, achieving an optimal PCE of 2.70%, with a Voc of 1.29 V, Jsc of 4.60 mA/cm^2^, and FF of 48.58%. In addition, after being stored for 500 h in air with an RH of 25–65% and a temperature of 25 °C, the unencapsulated optimal PSCs still retained 69% of their initial PCE. This demonstrates that BP, used as a hole transport layer, can effectively enhance the stability of PSCs, indicating a promising application potential for BP materials in practical use. However, the instability of BP in the air and its susceptibility to oxidation limit its application in solar cells. Improving the air stability of BP remains a key issue for its widespread use. Additionally, the poor phase stability and higher bandgap of the CsPbBr_3_ crystal structure result in significantly lower cell efficiency compared to organic–inorganic hybrid PSCs. How to optimize the interaction between BP and CsPbBr_3_, reduce the density of defect states, and enhance the stability of CsPbBr_3_ PSCs continue to pose a major challenge for BP-based all-inorganic perovskite solar cells.

## Figures and Tables

**Table 1:** Photovoltaic parameters (Voc, Jsc, FF, and PCE) of CsPbBr_3_ PSC with different c-TiO_2_ spin-coating times, different m-TiO_2_ spin-coating times, different BP concentrations, and different BP spin-coating times.

Sample	Voc (V)	Jsc (mA/cm^2^)	FF (%)	PCE (%)
1c-TiO_2_	1.18	3.36	40.55	1.61
2c-TiO_2_	1.21	4.15	45.98	2.31
3c-TiO_2_	1.20	3.56	39.73	1.70
4c-TiO_2_	1.18	3.31	36.68	1.43
1m-TiO_2_	1.21	4.15	45.98	2.31
2m-TiO_2_	1.21	3.25	40.96	1.61
3m-TiO_2_	1.19	2.47	38.02	1.12
4m-TiO_2_	1.21	2.22	39.86	1.07
0.2 mg/mL BP	1.16	4.15	39.45	1.90
0.4 mg/mL BP	1.17	4.73	43.70	2.42
0.6 mg/mL BP	1.13	3.28	40.25	1.49
0.8 mg/mL BP	1.12	3.13	40.02	1.40
1-BP	1.29	4.60	45.52	2.70
2-BP	1.26	3.80	40.02	1.91
3-BP	1.16	3.67	42.56	1.81
4-BP	1.15	3.57	39.71	1.63

**Table 2:** Photovoltaic parameters (including Voc, Jsc, FF, and PCE) of CsPbBr_3_ PSCs with 0.4 mg/mL BP as the HTL and without HTL, under a light intensity of 100 mW/cm^2^.

Sample	Voc (V)	Jsc (mA/cm^2^)	FF (%)	PCE (%)
Without HTL	1.22	2.65	39.51	1.27
With BP	1.15	3.57	39.71	1.63

**Table 3:** TRPL spectral parameters of CsPbBr_3_ thin films without HTL and CsPbBr_3_/BP thin films.

Structure	τ_1_	τ_2_
Without HTL	4.931	23.961
with BP	4.055	14.337

**Table 4:** EIS parameters of CsPbBr_3_ PSC with 0.4 mg/mL BP as HTL and without HTL.

Sample	Rs (Ω)	Rrec (Ω)	CPE
Without HTL	91.37	12,397	9.554 × 10^−9^
With BP	22.75	28,862	6.798 × 10^−9^

## Data Availability

The original contributions presented in the study are included in the article, further inquiries can be directed to the corresponding author.
